# uap: reproducible and robust HTS data analysis

**DOI:** 10.1186/s12859-019-3219-1

**Published:** 2019-12-12

**Authors:** Christoph Kämpf, Michael Specht, Alexander Scholz, Sven-Holger Puppel, Gero Doose, Kristin Reiche, Jana Schor, Jörg Hackermüller

**Affiliations:** 1 Young Investigators Group Bioinformatics and Transcriptomics, Department Molecular Systems Biology, Helmholtz Center for Environmental Research – UFZ, Permoserstraße 15, Leipzig, 04318 Germany; 2 Bioinformatics Department, Universität Leipzig, Härtelstraße 16-18, Leipzig, 04107 Germany; 3 Bioinformatics Unit, Department of Diagnostics, Fraunhofer Institute for Cell Therapy and Immunology, Perlickstraße 1, Leipzig, 04103 Germany; 4 Present address: Department of Diagnostics, Fraunhofer Institute for Cell Therapy and Immunology, Perlickstraße 1, Leipzig, 04103 Germany; 5 Present address: ecSeq Bioinformatics GmbH, Sternwartenstraße 29, Leipzig, 04103 Germany

**Keywords:** Sequencing data analysis, Work-flow management, Reproducible research

## Abstract

**Background:**

A lack of reproducibility has been repeatedly criticized in computational research. High throughput sequencing (HTS) data analysis is a complex multi-step process. For most of the steps a range of bioinformatic tools is available and for most tools manifold parameters need to be set. Due to this complexity, HTS data analysis is particularly prone to reproducibility and consistency issues. We have defined four criteria that in our opinion ensure a minimal degree of reproducible research for HTS data analysis. A series of workflow management systems is available for assisting complex multi-step data analyses. However, to the best of our knowledge, none of the currently available work flow management systems satisfies all four criteria for reproducible HTS analysis.

**Results:**

Here we present uap, a workflow management system dedicated to robust, consistent, and reproducible HTS data analysis. uap is optimized for the application to omics data, but can be easily extended to other complex analyses. It is available under the GNU GPL v3 license at https://github.com/yigbt/uap.

**Conclusions:**

uap is a freely available tool that enables researchers to easily adhere to reproducible research principles for HTS data analyses.

## Background

Next generation or high throughput sequencing (HTS) methods that rely on massively parallel DNA sequencing have opened a new era of molecular life sciences. A continuous growth in sequencing throughput, precision, length of the sequencing reads, and increasing automation and miniaturization led to a huge advantage in manual efforts and costs per experiment compared to traditional Sanger sequencing. HTS has thus become a mainstay in many fields of biology and biomedicine: Apart from de novo genome sequencing, HTS is not only increasingly replacing other massively parallel techniques like microarrays in transcriptomics or epigenomics, but also allows for new approaches e.g. in microbial ecology, population genetics, clinical diagnostics, or breeding. As a consequence, the amounts of HTS-generated data are exploding in many disciplines, associated with challenges for storage, transfer, curation and particularly reproducible analysis of these data [1].

HTS data analysis is a multi-step process and fairly complex compared to the analysis of other biological data. Analysis of e.g. gene expression microarray data is involving image processing and statistical analysis of the expression signal. Analysis of a comparable transcriptome sequencing (RNA-seq) data set involves – apart from the initial base calling – at least clipping of adapter and low quality sequences, mapping to a reference genome, counting of mapped reads per annotation element and statistical analysis and may include many other steps like transcript de novo assembly or analysis of differential splicing. For most of these steps a selection from a range of available bioinformatic tools needs to be made and for most tools a variety of parameters needs to be set.

**Fig. 1:**
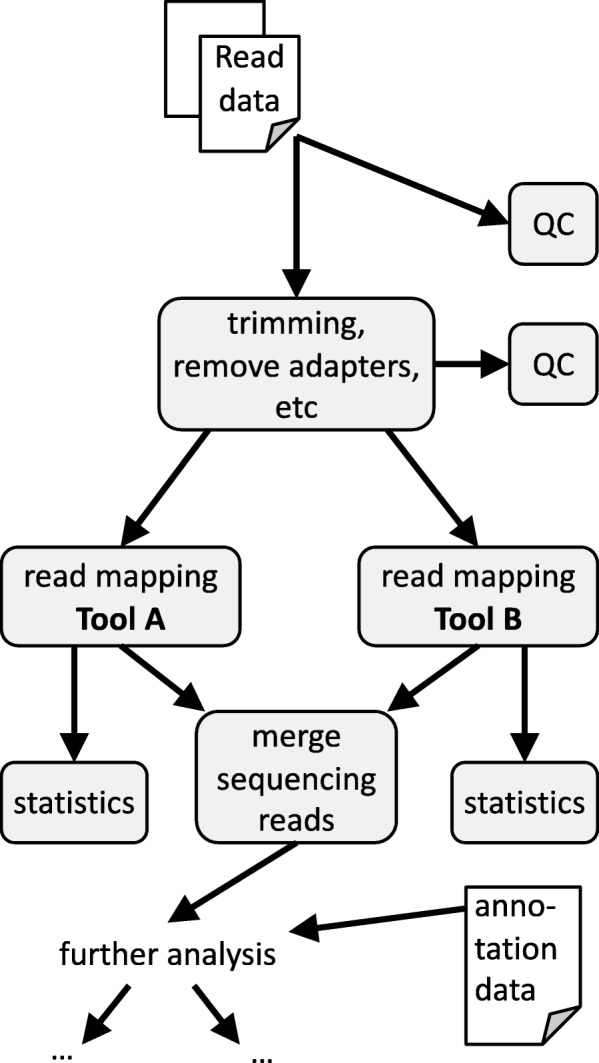
Sketch of a prototypic DAG describing the analysis of unmapped HTS reads. HTS data analysis typically follows a DAG-like structure. Nodes of the DAG are called steps and may depend on preceding steps and/or branch out to subsequent parts of the analysis. Results of individual steps may be merged again in later steps and processed further

In general, an HTS data analysis can be described as a directed acyclic graph (DAG) like structure. We call a node in the DAG a step. Steps may depend on previously computed results (produced by preceding steps) and/or branch out to subsequent parts of the analysis. Results of individual steps also may be merged again in later steps and processed further. See Fig. 1 for a sketch of a prototypic HTS analysis.

Independent replication is a fundamental principle for evaluating published findings. If a complete replication is not feasible e.g. due to the access to samples or cost and effort for HTS experiments, reproducing the analysis from raw data to published claims is the second best alternative [2]. The degree of reproducibility of biomedical research has been criticized [3] and a ”reproducibility crisis” has been diagnosed recently [4]. Given the complexity of HTS data analyses outlined above, reproducibility is particularly dependent on a detailed reporting of analysis details. However, a critical analysis of published HTS-based genotyping studies revealed that less than a third of the studies analyzed provided sufficient information to reproduce the mapping step [1].

Several appeals have been made to alleviate these reproducibility issues in computer science and computational biology. Roger Peng emphasized the necessity of linking executable analysis code and data as the gold standard second to full replication [2]. Sandve and colleagues called for adhering to ”ten simple rules for reproducible computational research”, which fully apply to HTS data analysis [5]. Finally, Grüning and coauthors defined a technology stack for reproducible research and formulate guidelines that particularly consider the numerical reproducibility of computation in the life sciences [6].

In our opinion, a minimal degree of reproducible research in managing HTS data analyses requires a tool which ensures that (*i*) the dependencies between analysis steps and intermediate results are correctly maintained, (*ii*) analysis steps are successfully completed prior to execution of subsequent steps, (*iii*) all tools, their versions and full parameter sets (including standard parameters which are usually not set when starting the tool from the commandline) are logged, (*iv*), the consistency between the code defining the analysis and the currently available results is ensured.

A series of different bioinformatic workflow management systems (WMS) is available to support complex DAG-like analyses. WMS that are appropriate for HTS analyses are in part general purpose systems, in part specific for HTS or even designed to address individual aspects of HTS data analysis. Also, different WMS designs require various levels of experience from the users, while providing different degrees of flexibility. WMS approaches like Ruffus [7] or SnakeMake [8] allow the implementation of highly individual analyses, either via domain-specific programming languages or a general-purpose programming language. iRAP [9], RseqFlow [10], or MAP-RSeq [11] belong to a group of WMS that implement a single specific type of HTS analysis. Several WMS encapsulate the individual steps of an analysis within modules and allow for their free combination, e.g. Galaxy [12], Unipro UGENE [13], KNIME [14], or Taverna [15] – many of which come with a graphical user interface. Finally, a group of lightweight modularized WMS aims at a modular, customizable, command-line-based approach, which includes e.g. bcbio-nextgen [16], Bpipe [17], or nextflow [18]. Several of these WMS rely on logging detailed information of used tools’ versions and issued commands. However, to the best of our knowledge, none of the published WMS satisfies all four criteria that we defined for maintaining a minimal degree of reproducible research in HTS data analysis. We compared the essential features, particularly regarding reproducibility, of a set of actively maintained, flexible, and modular WMS in more detail (Table 1, Additional file 1: Table S1).

**Table 1:** Comparison of workflow management systems and HTS analysis pipelines based on essential features, particularly regarding reproducibility

	

Tools were selected from https://github.com/pditommaso/awesome-pipelineretaining only tools that were considered to be actively developed by at least five contributors (latest commit after 31.01.2018), are licensed as open source, where the scope of application is clearly on bioinformatics, and which support cluster batch systems. **Modular**: Workflows are assembled from reusable steps; **Customizable**: Configuration of the analysis is separated from the code defining the steps; **Flexible**: Workflows can easily be altered without programming skills; **Failure recovery**: Failure during execution is detected and subsequent analyses halted to avoid corrupted results; **Reproducibility - Dependencies**: WMS enforces that dependencies between analysis steps and intermediate results are correctly maintained. **Consistency**: WMS safeguards that analysis steps are successfully completed prior to execution of subsequent steps. **Linking code and result**: WMS ensures consistency between the code defining the analysis and the currently available results. **Logging**: WMS logs Stdout/Stderr, exit status, tool versions, WMS version, executed commands, execution date, and in-/output files (see corresponding Additional file 1: Tables S1 and S2 for details); **Data authenticity** WMS records information about creator and creating process(es) of the data; **Data integrity**: WMS records information that allows to verify the integrity of created data (e.g. hash sums); **Supp. platforms - Cluster**: WMS supports compute cluster management systems ton run jobs; **Docker**: WMS can run jobs using Docker images; **Cloud**: WMS can be deployed in a compute cloud; **Supp. CWL**: WMS supports common workflow language; **Local**: WMS can be executed locally without depending on a cluster management system or a (web)server; Results are given as ◯ (not met), ◖◯ (partially met), ● (fully met) and – (not stated). Ratings are based on information provided in the papers, documentations and manuals. A more detailed comparison is presented in Additional file 1: Table S1

Here, we introduce the workflow management system uap (Universal Analysis Pipeline) that may be used to implement any DAG-like data analysis workflow, but is primarily aimed at HTS data analysis. It is constructed to execute, control, and keep track of the analysis of large data sets. uap encapsulates the usage of (not necessarily bioinformatic) tools and handles data flow and processing of the complete analysis. Produced data is tightly linked with the code specifying the analysis. Thus, it enables users to perform reproducible, robust, and consistent data analyses. We provide complete workflows for handling genomic data and analyzing RNA-Seq and Chromatin Immuno-Precipitation DNA-Sequencing (ChIP-seq) data, which can be used as templates that allow for easy customization. As we are also integrating steps for downloading published sequencing raw data (e.g. from SRA), uap enables users to efficiently reproduce the data analysis of published studies. The provided workflows have been optimized for minimal I/O load on high performance computing (HPC) environments. Although, initially designed for HTS data analysis, the plugin architecture of uap allows for the expansion to any kind of data analysis.

## Implementation

uap is a workflow management software (WMS) implemented in Python. It provides user-friendly access to a range of bioinformatic analyses of large datasets, such as high throughput sequencing data. Each analysis is completely described by an individual configuration file in YAML^1^ format. The steps of the analysis and their dependencies as well as the required tools, parametrization and locations in the file system are specified there. Based on these settings uap constructs a directed acyclic graph (DAG) that represents the workflow of the analysis.

**Analysis as a directed acyclic graph:** The DAG represents single analysis steps as nodes and pairwise dependencies between steps as directed edges. A *step* is a blueprint for a particular analysis with defined input and output data. The passing of input data to a step and the generation of a particular type of output data is modeled as *connections*. They control the data flow between steps by grouping output files and providing them to down-stream steps. uap distinguishes between source and processing steps. *Source steps* emit data into the workflow and hand the files over to downstream processing steps via output connections. The user is free to categorize the input data files for a workflow into user specified groups to create separate output locations for each category. *Processing steps*, on the other hand, receive data from upstream steps via input connections, define a sequence of execution commands and assign output file locations for each input connection. The entirety of these configurations of a step for a particular set of input connections is called a *run*. It can be interpreted as an instance of a step and is the atomic unit of the analysis. Additional file 1: Figure S1 shows the DAG including its runs rendered by uap based on the configuration file for the analysis of a published data set.

**Plug-in architecture:** Steps encapsulate the usage of a tool in a single python class, which allows users to easily customize uap by adding steps. Every new step inherits from a super class, defines incoming and outgoing connections, the required tools, and has to implement the runs() method. A step can individually be optimized for efficient use of CPU and memory usage. For allowing a flexible accommodation to different high performance computing environments, uap supports a step-specific adaptation of the environment, e.g. for setting variables, or automatic loading and unloading of software modules. Additional file 1: Figure S3 sketches how new processing steps are defined.

**Enforcing consistency and integrity:** When computing on large data sets, partial processing of large files due to premature termination of tools may remain undetected without stringent monitoring of processes and poses a severe threat to data integrity. In uap, runs are therefore executed in a temporary directory and monitored throughout execution. The overall workflow is not compromised in case a single run fails. Result files are only moved to their final location if all processes of a run exited gracefully and all expected output files exist.

uap automatically re-schedules runs if it detects failed processes or missing files. Also, changes in the configuration trigger re-scheduling of the affected runs and all dependent runs in the DAG.

**Maintaining reproducibility:** uap tightly links analysis code and resulting data by hashing over the complete sequence of commands including parameter specifications of a run and appending the key to the output path. Thus, any changes to the analysis code alter the expected output location, which allows uap to check whether analysis code and output correspond to each other.

At execution time, an annotation file in YAML format is captured for each run that contains the complete content of the configuration at this point. Hence, an executed run is documented with the releases of all used software and the invoked command line with all parameter settings. In addition, memory and CPU usage of each process, checksums of result files, as well as the last kB of stdout and stderr output are reported. The annotation file is stored next to the result files of a run.

**Fig. 2:**
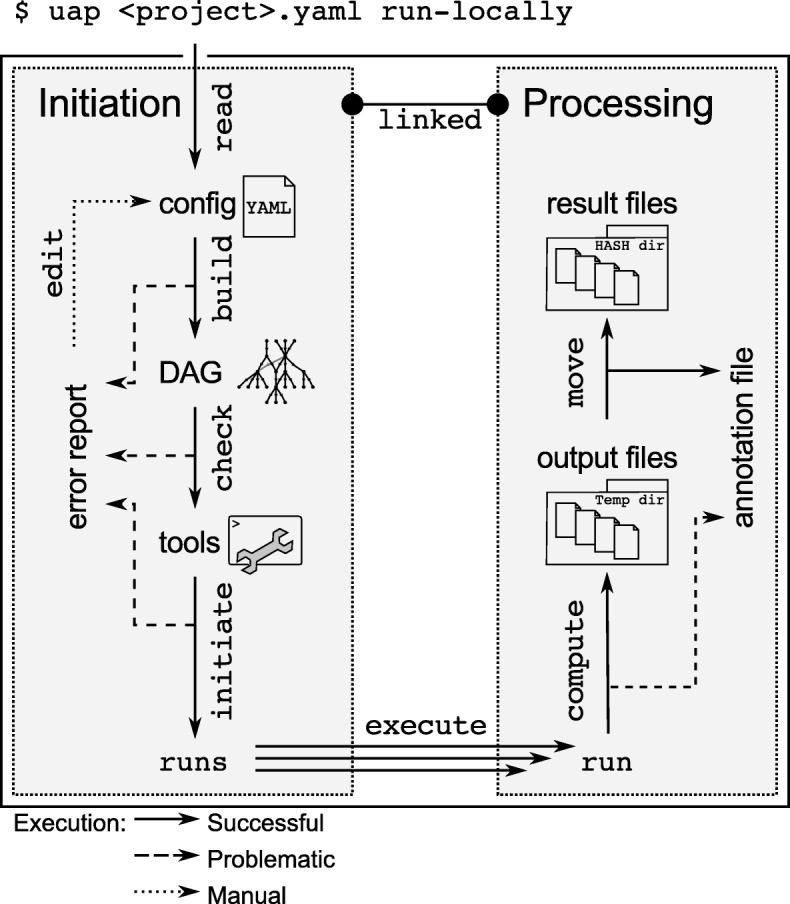
uap’s process flow, error reporting, and the link between the analysis code and result files. uap implements a failing fast approach: the DAG is built from the configuration file, tested to be acyclic, all required tools are tested for their availability and the status of all steps is determined. Subsequently, uap can start runs, display the commands of runs, show the state of the runs, and render execution graphs. Runs are executed in temporary directories and monitored throughout execution. Result files are only generated at their final location if all processes of a run exited gracefully and all expected output files exist. Analysis code and resulting data are tightly linked by hashing over the complete sequence of commands and parameters of a run and appending the key to the output path. Each run generates an annotation file in YAML format that captures the configuration, software versions and releases, the invoked command line, all parameters, memory and CPU usage of each process involved, checksums of the result files, as well as the last kB of stdout and stderr

**Process flow:** Initially, uap reads the configuration, generates the respective DAG, and defines all commands and output file names. Throughout this initiation process uap inspects the planned analysis for potential errors. The graph is tested to be acyclic, all required tools (in defined releases) are tested for their availability and the status of all steps is determined. This initiation phase is executed early, i.e. before submitting runs to a compute cluster. uap thus implements a failing fast technique. This is an important feature when working with large amounts of data on HPC systems where software is dynamically loaded and erroneous configurations might otherwise only become apparent after hours of computation. Figure 2 illustrates uap’s process flow, error reporting, and the link between configuration and result files.

**Fig. 3:**
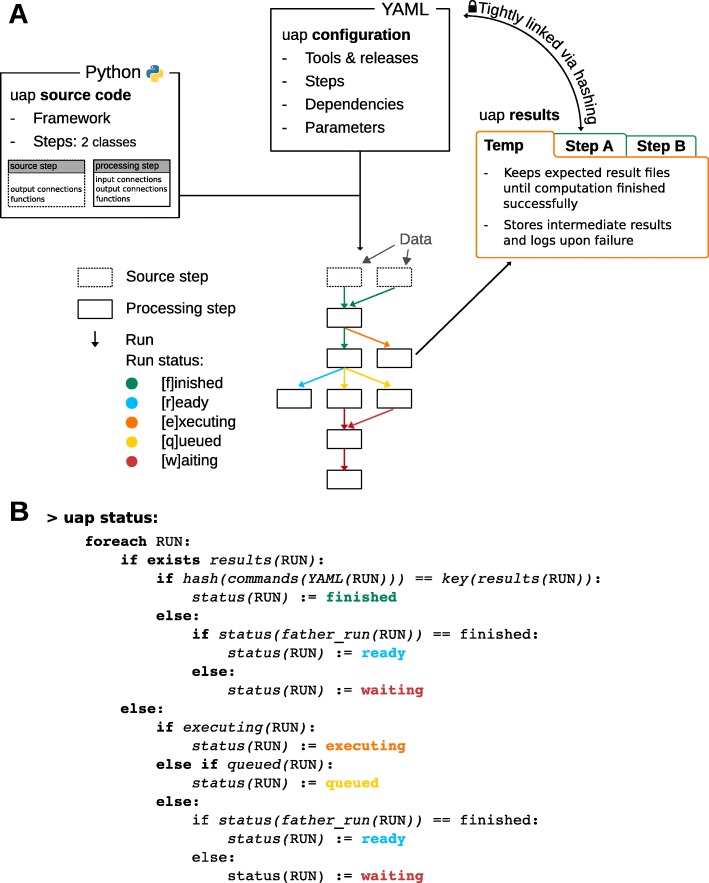
Sketch of the main principles uap is built on. **a** An analysis with uap comprises three parts: (i) the uap **source code** itself, implemented in Python - it contains the complete framework of uap and 2 classes for the implementation of source and processing steps. These classes are used to wrap any tool that is part of the analysis, enabling an easy extension of the uap’s repertoire of steps; (ii) the uap **configuration** in YAML format. It contains all necessary information to run and reproduce the analysis given the data; and (iii) the uap **results** - organized in one folder per step in the output directory. The special folder *temp* contains the expected results until the computation of the step has finished successfully, and keeps the intermediate results and log files upon failure. **b** The progress or state of an analysis can be monitored with a call to uap status. It determines the state of each individual step in dependence of the state(s) of its previous step(s) and provides this information to the user

Subsequently, uap can start runs, display the commands of runs, show the state of the runs, and render execution graphs. Execution graphs are useful tools to inspect the performance of an analysis, e.g. to identify resource bottlenecks in a pipeline of commands. Additional file 1: Figure S2 shows such an execution graph. Figure 3 provides an overview of the main principles uap is built on.

## Results

uap is a workflow management system dedicated to data consistency and adoption of a Reproducible Research paradigm in HTS data analysis. uap runs on UNIX-like operating systems and can interact with batch queuing systems like the Sun/Oracle/Univa grid engines (SGE/OGE/UGE) and SLURM [23] to submit analyses to high performance computing systems. uap is distributed under the GNU GPL v3 license and is publicly available at https://github.com/yigbt/uap. Its documentation is hosted at http://uap.readthedocs.org/. A docker container with a core set of tools is available at https://hub.docker.com/r/yigbt/uap/tags

uap is distributed with predefined workflows for (*i*) genome sequence download and index generation for read mapping programs, (*ii*) transcriptome sequencing (RNA-seq) data analysis, and (*iii*) Chromatin Immuno-Precipitation DNA-Sequencing (ChIP-Seq) data analysis. Further, we provide small test data sets enabling a quick start for each of these workflows. An additional example using a larger data set is provided via code for downloading and analyzing a publicly available ChIP-Seq data set (Barski et al. [24]). The provided workflows are intended to serve as an easy entry point into a uap analysis as well as a template for similar analyses, e.g. for other species or with another set of tools.

**Preparing genomic data for HTS analysis:** An important prerequisite for HTS projects where a reference genome is available is aligning (mapping) the sequencing reads to this genome. Most mapping software requires a specific data structure (index) of the genome to efficiently solve this alignment problem. Indices have to be generated once, prior to any mapping procedure. We provide uap configuration files for the bwa [25], bowtie [26] and segemehl [27] mapping programs and samtools (fasta indexing) of the a) *Mycoplasma genitalium*, and b) *Homo sapiens* genome. Genomic sequences can be downloaded automatically prior to index generation.

**Transcriptome assembly from RNA-Seq data:** Transcriptome sequencing identifies and estimates the quantity of RNA in biological samples. Beyond quantification of known transcripts based on overlapping sequence reads, RNA-seq allows the assembly of novel transcripts. We provide a uap configuration file for combining split-read mapping with de novo transcript assembly. uap reads the sequencing data either from an Illumina sequencing run folder, or a set of fastq files, applies quality control, removes adapter sequences, and maps the reads to a genome using tophat2 [28] and segemehl [27, 29]. The mapped reads from tophat2 are directly processed by cufflinks [30] for de novo transcript assembly. Split-reads mapped with segemehl are prepared for cufflinks using an adapter script and then also processed with cufflinks. The configuration also contains a step to determine the number of mapped reads per transcript applying htseq-count [31].

**Identification of enriched regions from ChIP-Seq data:** ChIP sequencing is a method that integrates chromatin immunoprecipitation (ChIP) and high-throughput DNA sequencing to identify sites of protein-DNA interactions. The provided uap-configuration file for this task initially resembles the RNA-Seq workflow. Here, reads are expected to correspond to genomic DNA and the mapping is done without considering split-reads using bowtie2 [26]. Mapped reads are subsequently sorted, duplicates removed, and enriched regions are detected using MACS2 [32].

## Conclusions

The critique on a lack of reproducibility in science and a growing awareness that many reported facts do not seem to hold up to repeated investigation has meanwhile reached a broader audience beyond the scientific community (e.g. [33–35]). In our opinion, HTS data analysis is particularly prone to consistency and reproducibility issues – especially due to the complexity of the analysis, the involved data volume, and the broad range of available tools and their multitude of parameters.

Workflow management systems are indispensable for reproducibly controlling more complex analyses, like HTS data analysis. Published workflow management systems for HTS data analysis are either highly flexible and made for experienced programmers or lack a lot of flexibility but can be used intuitively and some are specific to a certain type of analysis. In the introduction we listed four minimal requirements that we consider essential for ensuring reproducibility and consistency of HTS data analyses. Additionally, but beyond the programmatic control of a of a WMS, using versioned external data like genome sequences or annotation is key for reproducible analysis. None of the published systems we are aware of, however, completely satisfies these criteria. uap has been designed to fulfill these. One critical requirement is linking analysis code and generated data. While *Reproducible Research* in statistics [36] uses tools like Sweave [37], knitR [38], or Jupyter [39] to combine analysis code and resulting data in one output file, such a strategy is not feasible for most steps of an HTS analysis due to the size of generated data. uap therefore relies on hashing over the complete sequence of commands including parameters of a run and appending the key to the output path. In addition, uap performs logging and process monitoring, supports different cluster management systems, creates recovery points, plots execution graphs, manages job dependencies, and is extensible to any kind of multi-step analysis. It provides pre-built steps for the preparation of genomic data and the analysis of RNA-Seq and ChIP-Seq data.

Among the many different flavors of WMS uap is clearly easier to operate for users with limited programming experience than systems based on domain specific programming languages, while offering a lot more flexibility than single purpose tools. Based on the comparison of tools in Additional file 1: Table S1, the tools most dedicated to reproducible research and providing the most similar set of features compared to uap are Galaxy and Nextflow.

In our opinion, Galaxy [40] and uap address different user groups and tasks and we use both WMS in our research environment. Galaxy is great for providing predefined workflows to users without experience in programming and working on the command line. It allows these users to adapt parameters and execute such workflows on their data. For users working frequently with large HTS data sets, adapting workflows to a larger extent or duplicating branches of the DAG for performing variants of the analysis in parallel is much more efficiently performed in uap. Galaxy does not link data and code in the sense of uap. But as any change to parameters in a Galaxy workflow triggers re-execution of the sub-workflow below, this is not necessary. If many changes throughout a workflow have to be made, this behavior of Galaxy may be hindering. Obviously, running HTS analyses on Galaxy requires a Galaxy server integrated with an HPC environment, which is not trivial to set up and demands continuous maintenance. Starting from scratch, setting up HTS analysis is significantly less effort using uap than Galaxy.

Nextflow is a powerful WMS dedicated to scalability and reproducibility [18]. Its approach to reproducibility relies on a tight integration with github and the support of scalable containerization of pipelines using e.g. Docker. Nextflow and uap share several concepts, e.g. using temporary files for intermediate results or analyzing the workflow DAG to enable failing fast. Intermediate results of an HTS analysis can consume large amounts of storage space. uap therefore provides a means for volatilization of intermediate results without breaking dependencies in the DAG - a feature which does not seem to be available in Nextflow. Logging is somewhat limited in Nextflow compared to uap, but Nextflow provides a broader support for HPC environments including also support for cloud computing. Nextflow’s approach to reproducibility is powerful when software from Github is used, as it enables the user to request a specific commit, or when the tools used are publicly available as a container. However, when a tool is run in ’native task support’ like the Kallisto example provided in [18], uap is more stringent in logging version and the full set (including default) of parameters. Where uap and Nextflow differ most clearly regarding reproducibility is linking data and code, as to our understanding based on publications and the online documentation this is not available in Nextflow.

In summary, we are convinced that reproducible research principles need to be advanced for HTS data analysis and that uap is a highly useful system for facing this challenge.

## Availability and requirements

**Project name:** uap **Project home page:**https://github.com/yigbt/uap**Operating system(s):** Linux. **Programming language:** Python. **Other requirements:** virtualenv, git, and graphviz. **License:** GNU GPL v3. **Any restrictions to use by non-academics:** None.

## Supplementary information

**Additional file 1** This file includes supplemental tables and figures.

## Data Availability

Source code is available at https://github.com/yigbt/uap, links to Docker containers and documentation are provided at https://www.ufz.de/index.php?en=44919. All datasets used within the example workflows are publicly available and are fully referenced within the workflow configuration files.

## Abbreviations

ChIP-seq: Chromatin immuno-precipitation dna-sequencing
DAG: Directed acyclic graph
HTS: High throughput sequencing
HPC: High performance computing
RNA-seq: Transcriptome sequencing
WMS: Workflow management system

## Footnotes

1 http://yaml.org/
