# Supplementary material for: uap: reproducible and robust HTS data analysis
Source: BMC Bioinformatics. 2019 Dec 12;20:664. doi: 10.1186/s12859-019-3219-1 (PMC6909466; doi:10.1186/s12859-019-3219-1)
Supplement: Supplementary file 1 — Additional file 1 This file includes supplemental tables and figures. [file 12859_2019_3219_MOESM1_ESM.pdf]

Supplemental material: uap: Reproducible and Robust HTS Data Analysis

November 11, 2019

List of Figures

Figure S1 DAG of the analysis of ChIP-seq data from Barski *et al.*. 4
Figure S2 Rendered graph of a finished `sam_to_sorted_bam` run. 5
Figure S3 Extending `uap`. 6

List of Tables

Table S1 Comparison of workflow management systems based on essential features, particularly regarding reproducibility. 2
Table S2 Availability of software tools used as pipeline and/or work-flow managers. 3

Table S1: **Comparison of workflow management systems based on essential features, particularly regarding reproducibility.** Tools were selected from `https://github.com/pditommaso/awesome-pipeline` retaining only tools that were considered to be actively developed by at least five contributors (latest commit after 31.01.2018), are licensed as open source, where the scope of application is clearly on bioinformatics, and which support cluster batch systems.
Modular: Workflows are assembled from reusable steps; **Flexible**: Configuration of analysis is separated from its code (code $\neq$ definition) and workflows can easily be adapted without programming skills; **Failure recovery**: Failure during execution is detected and analyses halted to avoid corrupted results; **Reproducibility - Dependencies**: WMS enforces that dependencies between analysis steps and intermediate results are correctly maintained. **Consistency**: WMS safeguards that analysis steps are successfully completed prior to execution of subsequent steps. **Linking code and result**: WMS ensures consistency between the code defining the analysis and the currently available results. **Logging**: WMS logs the mentioned information; **Data authenticity** WMS records information about creator and creating process(es) of the data; **Data integrity**: WMS enables validating the pristine state of created data, e.g. using hash sums; **Supp. platforms**: WMS is designed to work with these technologies; **Docker**: WMS can run jobs using Docker images **Supp. CWL**: WMS supports CWL;
Locally executable: WMS can be without cluster or (web)server; Results are given as ○ (not met), ◐ (partially met), ● (fully met) and – (not stated). In case of supported platforms, ● corresponds to running out of the box, while ◐ indicates that the WMS can be used on the respective platform upon adaptataions of the configuration or extension of code. Ratings are based on information provided in the papers, documentations and manuals. Details on the availability of these tools are provided in Supplemental Table S2.

	Arvados	bcbio-nextgen[1]	BigDataScript[2]	Bpipe[3]	Cluster Flow	Cosmos	Cromwell	Galaxy	Gwf	Hive	Nextflow	PypeFlow	Rabix	Toil	uap
Modular	◐	○	◐	●	●	◐	●	●	◐	●	◐	–	◐	◐	●
Flexible	●	●	○	◐	●	○	●	●	○	◐	●	–	●	●	●
Failure recovery	–	–	●	●	–	●	●	●	–	–	●	–	◐	●	●
Reproducibility															
Dependencies	–	–	◐	◐	–	○	●	●	–	–	●	–	–	–	●
Consistency	●	●	●	●	●	●	●	●	●	●	●	●	●	●	●
Linking code and result	–	–	–	–	–	–	–	●	–	–	●	–	–	–	●
Logging															
Stdout/Stderr	–	●	●	●	●	–	●	●	◐	–	◐	–	–	◐	●
Exit status	●	–	●	●	–	–	●	●	–	–	●	–	–	–	●
Tool versions	–	–	–	◐	◐	–	–	●	–	–	–	–	–	–	●
WMS version	●	–	–	–	–	–	–	–	–	–	●	–	–	–	●
Executed commands	●	●	●	●	◐	●	–	●	–	–	●	–	–	–	●
Execution date	●	–	–	●	–	●	–	●	–	○	●	–	–	–	●
In-/output files	●	–	–	●	●	◐	–	●	–	–	–	–	–	–	●
Data authenticity	●	–	–	–	–	●	–	●	–	–	–	–	–	–	●
Data integrity	●	–	–	–	◐	○	–	○	–	–	–	–	●	–	●
Supp. platforms															
SGE/OGE/UGE	○	●	●	●	●	–	●	●	●	●	●	◐	◐	●	●
SLURM	●	●	●	○	●	●	●	●	●	○	●	◐	◐	●	●
Torque	○	●	○	○	○	–	◐	●	○	●	●	◐	◐	●	◐
Moab	○	–	●	○	○	–	◐	○	○	○	○	○	◐	–	◐
LSF	○	●	◐	●	●	●	●	●	○	●	●	◐	◐	●	◐
Cloud (EC2)	◐	◐	◐	–	–	●	◐	●	–	◐	●	–	●	●	○
Docker	●	●	–	–	–	–	●	●	–	●	●	–	●	●	○
Supp. CWL	●	◐	–	○	○	○	●	●	○	○	●	○	●	●	○
Locally executable	○	●	●	●	●	●	●	◐	●	●	●	–	●	●	●

Table S2: **Availability of software tools used as pipeline and/or work-flow managers.**

Tool	Reference	url
Arvados		https://arvados.org/
bcbio-nextgen	[1]	https://github.com/bcbio/bcbio-nextgen
BigDataScript	[2]	https://pcingola.github.io/BigDataScript/
Bpipe	[3]	https://github.com/ssadedin/bpipe
Cluster Flow	[4]	http://clusterflow.io
Cosmos	[5]	https://cosmos.hms.harvard.edu
Cromwell		https://github.com/broadinstitute/cromwell
Galaxy	[6]	https://usegalaxy.org/
Gwf		https://github.com/gwforg/gwf
Hive		https://github.com/Ensembl/ensembl-hive
Nextflow	[7]	https://www.nextflow.io/
PypeFlow		https://github.com/PacificBiosciences/pypeFLOW
Rabix	[8]	https://github.com/rabix/rabix
Toil		https://github.com/DataBiosphere/toil
uap		https://github.com/yigbt/uap

Figure S1: **DAG of the analysis of ChIP-seq data from Barski *et al.* [9]**. Yellow boxes contain the names of the steps and, if step name and type differ, additionally the type of the step in brackets. The adjacent, attached boxes display the number of finished versus total runs. The color coding of the latter boxes indicates whether that step has been finished (green), partially finished (ocher), or not started yet (red).

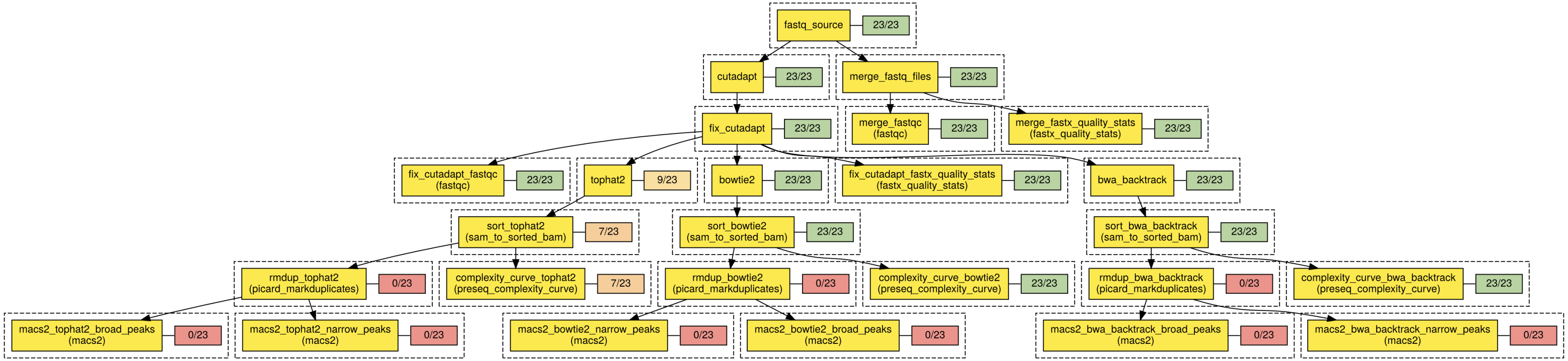

Figure S2: **Rendered graph of a finished `sam_to_sorted_bam` run.** The graph was created by `uap`'s `render` subcommand and manually edited to better fit to the page. It shows CPU and memory usage of the run and its executed commands.

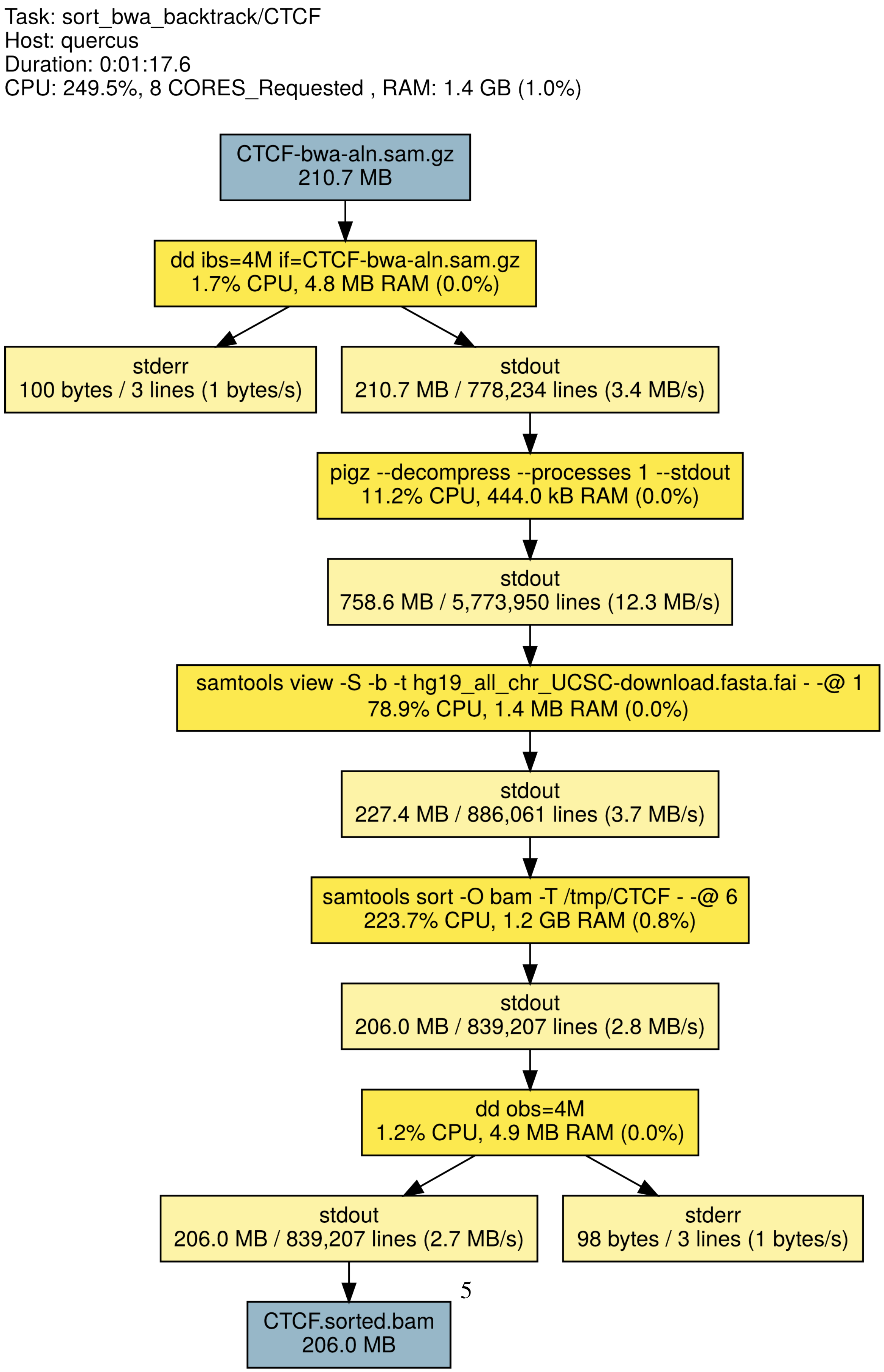

Figure S3: **Extending uap.** `uap` can be easily extended by implementing new source or processing steps. New steps need to define a new class that is derived from `AbstractStep` or `AbstractSourceStep`, respectively, and are added to uap by placing a single Python file into the steps or source steps folder. This sketch provides pseudocode for a new processing step that applies a `tool` on (a number of) input files and returns the reults in the respective (number of) output files. We describe this in detail at `https://uap.readthedocs.io/en/v1.0/extension.html`.

```python
import sys
import os
from logging import getLogger
from abstract_step import AbstractStep

logger = getLogger('uap_logger')


class a_new_step(AbstractStep):
    '''
    Provide documentation for a_new_step here.

    If a_new_step wraps a publicly available tool provide a link to the tool web page.
    https://some.url/software/a_new_step/index.shtml
    '''

    def __init__(self, pipeline):
        super(a_new_step, self).__init__(pipeline)

        ## The new class needs to be derived from either AbstractStep, for
        ## processing steps, or AbstractSourceStep, for source steps.

        ## Define the default number of cpu cores to be requested for this step
        self.set_cores(6)

        ## Add input and output file connections
        self.add_connection('in/text')
        self.add_connection('out/text')

        ## Define all required tools, the path to the tool needs to be
        ## specified in the configuration yaml.
        self.require_tool('a_tool')

        # Add all paramters/options of the tool, including default parameters.

        self.add_option('show-all', bool, optional=True,
                        description="Show all characters")

    def runs(self, run_ids_connections_files):
        ## Create a list of all set options for a_tool: option_list
        ### No shown here to keep the figure legible

        ## Iterate over all run IDs ...
        for run_id in run_ids_connections_files.keys():
            input_paths = run_ids_connections_files[run_id]['in/text']

            ## ... and declare a new run for each of them.
            with self.declare_run(run_id) as run:
                # Get list of input files
                an_input_file = run_ids_connections_files[run_id]['in/text']

                ## An exec_group is a list of commands which are executed in
                ## one go. You might create multiple exec_group's if you need
                ## to make sure a set of commands finished before another set
                ## is started.
                with run.new_exec_group() as exec_group:
                    ## Construct the command
                    a_new_step = [self.get_tool('a_tool')]
                    a_new_step.extend(option_list)
                    a_new_step.extend(an_input_file)

                ## Add command to the exec group
                exec_group.add_command(a_new_step,
                                       stdout_path=log_outfile,
                                       stderr_path=err_outfile)
```

References

[1] Guimera, R.V.: bcbio-nextgen: Automated, distributed next-gen sequencing pipeline. EMBnet.journal **17**, 30 (2012). doi:10.14806/ej.17.B.286

[2] Cingolani, P., Sladek, R., Blanchette, M.: BigDataScript: a scripting language for data pipelines. Bioinformatics **31**(1), 10–16 (2014). doi:10.1093/bioinformatics/btu595

[3] Sadedin, S.P., Pope, B., Oshlack, A.: Bpipe: a tool for running and managing bioinformatics pipelines. Bioinformatics (Oxford, England) **28**(11), 1525–6 (2012). doi:10.1093/bioinformatics/bts167

[4] Ewels, P., Krueger, F., Kller, M., Andrews, S.: Cluster flow: A user-friendly bioinformatics workflow tool. F1000Research **5**, 2824 (2016). doi:10.12688/f1000research.10335.2

[5] Gafni, E., Luquette, L.J., Lancaster, A.K., Hawkins, J.B., Jung, J.-Y., Souilmi, Y., Wall, D.P., Tonellato, P.J.: Cosmos: Python library for massively parallel workflows. Bioinformatics (Oxford, England) **30**, 2956–2958 (2014). doi:10.1093/bioinformatics/btu385

[6] Afgan, E., Baker, D., Batut, B., van den Beek, M., Bouvier, D., Cech, M., Chilton, J., Clements, D., Coraor, N., Grning, B.A., Guerler, A., Hillman-Jackson, J., Hiltemann, S., Jalili, V., Rasche, H., Soranzo, N., Goecks, J., Taylor, J., Nekrutenko, A., Blankenberg, D.: The galaxy platform for accessible, reproducible and collaborative biomedical analyses: 2018 update. Nucleic acids research **46**, 537–544 (2018). doi:10.1093/nar/gky379

[7] Di Tommaso, P., Chatzou, M., Floden, E.W., Barja, P.P., Palumbo, E., Notredame, C.: Nextflow enables reproducible computational workflows. Nature biotechnology **35**, 316–319 (2017). doi:10.1038/nbt.3820

[8] Kaushik, G., Ivkovic, S., Simonovic, J., Tijanic, N., Davis-Dusenbery, B., Kural, D.: Rabix: An open-source workflow executor supporting recomputability and interoperability of workflow descriptions. Pacific Symposium on Biocomputing. Pacific Symposium on Biocomputing **22**, 154–165 (2017). doi:10.1142/9789813207813_0016

[9] Barski, A., Cuddapah, S., Cui, K., Roh, T.-Y., Schones, D.E., Wang, Z., Wei, G., Chepelev, I., Zhao, K.: High-resolution profiling of histone methylations in the human genome. Cell **129**(4), 823–37 (2007). doi:10.1016/j.cell.2007.05.009
